# Burden of prediabetes, undiagnosed, and poorly or potentially sub-controlled diabetes: Lolland-Falster health study

**DOI:** 10.1186/s12889-020-09791-2

**Published:** 2020-11-16

**Authors:** Neda Esmailzadeh Bruun-Rasmussen, George Napolitano, Allan Kofoed-Enevoldsen, Stig Egil Bojesen, Christina Ellervik, Knud Rasmussen, Randi Jepsen, Elsebeth Lynge

**Affiliations:** 1 Center for Epidemiological Research, Nykøbing Falster Hospital, Strandboulevarden 64, DK-4800 Nykøbing Falster, Denmark; 2 Department of Public Health, University of Copenhagen, Copenhagen, Denmark; 3 Department of Endocrinology, Nykøbing Falster Hospital, Nykøbing Falster, Denmark; 4 Department of Clinical Biochemistry, Herlev and Gentofte Hospital, Copenhagen, Denmark; 5 Data and Development Support, Region Zealand, Sorø, Denmark; 6 Department of Laboratory Medicine, Boston Children’s Hospital & Havard Medical School, Boston, MA USA

**Keywords:** Diabetes mellitus, Prediabetes, Undiagnosed diabetes, Glycaemic control, Lolland-Falster health study

## Abstract

**Background:**

This study aimed to investigate prevalence and risk factors for prediabetes, undiagnosed diabetes mellitus, poorly and potentially sub-controlled diabetes in a rural-provincial general adult population in Denmark.

**Methods:**

Using cross-sectional data from the Lolland-Falster Health Study, we examined a total of 10,895 individuals aged 20 years and above.

**Results:**

Prevalence of prediabetes was 5.8% (men: 6.1%; women: 5.5%); of undiagnosed diabetes 0.8% (men: 1.0%; women: 0.5%); of poorly controlled diabetes 1.2% (men: 1.5%; women: 0.8%); and of potentially sub-controlled diabetes 2% (men: 3.0%; women: 1.3%). In total, 9.8% of all participants had a diabetes-related condition in need of intervention; men at a higher risk than women; RR 1.41 (95% CI 1.26–1.58); person aged + 60 years more than younger; RR 2.66 (95% CI 2.34–3.01); obese more than normal weight person, RR 4.51 (95% CI 3.79–5.38); smokers more than non-smokers, RR 1.38 (95% CI 1.19–1.62); persons with self-reported poor health perception more than those with good, RR 2.59 (95% CI 2.13–3.15); low leisure time physical activity more than those with high, RR 2.64 (95% CI 2.17–3.22); and persons with self-reported hypertension more than those without, RR 3.28 (95% CI 2.93–3.68).

**Conclusions:**

In the Lolland-Falster Health Study, nearly 10% of participants had prediabetes, undiagnosed diabetes, poorly controlled, or potentially sub-controlled diabetes. The risk of these conditions was more than doubled in persons with self-reported poor health perception, self-reported hypertension, low leisure time physical activity, or measured obesity, and a large proportion of people with diabetes-related conditions in need of intervention can therefore be identified relatively easily.

**Supplementary Information:**

The online version contains supplementary material available at 10.1186/s12889-020-09791-2.

## Background

Diabetes mellitus (DM) is a metabolic disease characterized by high blood glucose levels resulting from defects in insulin secretion, insulin action, or both [1]. It is one of the most prevalent chronic diseases worldwide affecting more than 451 million adults in 2017 with a projected increase to 693 million by 2045 [2]. According to the International Diabetes Federation (IDF), more than 224 million adults with DM remain undiagnosed due to a combination of lack of awareness, poor health systems, slow onset of symptoms, and slow disease progression [2]. Undiagnosed, poorly or sub-controlled DM can cause lifelong complications, including kidney disease, heart disease, blindness and death, and undiagnosed DM is associated with a 1.5- to 3.0-fold mortality compared with no DM [3, 4]. A high-risk state for developing type 2 DM (T2DM) is prediabetes; where the blood glucose level is above normal but below the diagnostic level of DM.

Public health actions focusing on identification, treatment and follow-up are essential for preventing and reducing the human, social and economic consequences of DM. This requires reliable estimates of prevalence and risk groups, and it requires easily available information about how to identify risk groups.

In Denmark, the incidence and prevalence of diagnosed DM are monitored using nationwide registers [5]. Some estimates of prediabetes and undiagnosed DM have been derived from population-based surveys [6] however, to our knowledge, no study has focused on DM related conditions in rural areas. Our study aimed to provide data from Lolland-Falster, a disadvantaged rural-provincial part of Denmark with a life expectancy below the national average [7], and with health problems reported more frequently than in the rest of the country [8]. Using data from a population-based survey in Lolland-Falster [9], we focused on prevalence and risk factors of prediabetes, undiagnosed DM, poorly and potentially sub-controlled DM. Though clinically distinguishable, these conditions share the need for intervention in terms of initiation and/or regulation of treatment.

## Methods

### Study population

Data were derived from the rural-Danish Lolland-Falster Health Study (LOFUS), a prospective cohort study that aims to establish baseline information on the health status of the inhabitants of Lolland-Falster [9]. In this study, persons aged 18 and above were randomly selected from the Danish Civil Registration System and invited with their household members of all ages to participate. Invitations were sent to all persons of the invited households either by electronic or ordinary mail. Invited persons who agreed to participate completed web-based questionnaires prior to the physical examination at one of the stationary sites. Half-way through the data collection, the overall participation rate was found to be 34.1% (men: 31.9%, women: 36.4%). A detailed description of the study protocol [9] and information on the socio-economic determinants of participation [10] have been published previously. For this study, we included adults aged 20 years and above (*n* = 10,895) recruited from 2016 to 2019, excluding women reported to be pregnant.

### Self-reported and health examination data

From questionnaires, we used data on current self-perceived health, which was measured by one question with five response options ranging from excellent to very poor. One-item measures of self-rated general health are widely used in population studies and have shown independent associations with a variety of health outcomes including morbidity and mortality [11]. Leisure time physical activity was measured by one question where the respondents stated their level of physical activity in leisure time during the last 12 months in one of four response categories: (1) mainly sedentary activities (TV-watching, reading), (2) light physical activities ≥ 4 h per week (walking, bicycling, light gardening), (3) sports or other more vigorous activities ≥ 4 h per week (heavy gardening), (4) highly vigorous physical activity several times per week (heavy exercise or competitive sports). Because of very few participants in the highest category of leisure time physical activity *n* = 268, the variable was classified into: ‘low’- (category 1), ‘moderate’- (category 2), and ‘high’- (category 3 and 4). Information on dietary habits was obtained from responses to one question regarding self-assessment of general dietary habits.

Furthermore, we used data on self-reported diabetes, insulin medication, medication with other antidiabetic drugs, cardiovascular disease (stroke, atherosclerosis, angina pectoris, deep-vein thrombosis, hypertension) and medication (antihypertensive medication, heart medication, diuretics, cholesterol lowering medications, anticoagulants), alcohol consumption, and smoking habits. Two-thirds of LOFUS participants reported rare or no alcohol consumption, a percentage not compatible with sales statistics [12], and data were missing for 10%. We did therefore not use this information. From the health examination, we used data on measured height and weight.

### Blood and urine samples

Non-fasting blood samples were collected at three study centers, and analyzed at the Department of Clinical Biochemistry at Nykøbing Falster Hospital accredited by the standard ISO 15189. We used data on glycated hemoglobin (HbA1c), triglycerides, serum high-density lipoprotein cholesterol (HDL) and serum total cholesterol. Furthermore, we used data on urine albumin-to-creatinine ratio (UACR) from spot urine tests.

### Diabetes definition

No DM was defined as HbA1c levels below 42 mmol/mol and no self-reported diagnosis of DM or use of antidiabetic medication including insulin and other antidiabetic medications. Prediabetes was defined, according to Danish guidelines, as HbA1c levels in the range of 42 mmol/mol to 47 mmol/mol [13] and, no self-reported diagnosis of DM and/or use of antidiabetic medication.

We defined undiagnosed DM as HbA1c ≥ 48 and no self-reported diagnosis of DM and/or use of antidiabetic drugs, while diagnosed DM or DM-awareness was defined as reporting a DM diagnosis and/or use of antidiabetic medication. It was not possible to distinguish between type 1 DM (T1DM) and T2DM due to lack of information.

We explored glycaemic control measured by HbA1c in participants who reported having a DM diagnosis and/or use of antidiabetic medication by 1) using the American Diabetes Association (ADA) recommendation on a reasonable HbA1c-level for adults of < 53 mmol/mol [14], and by 2) dividing the diabetic population into three groups: poorly controlled DM (HbA1c ≥ 60 mmol/mol); potentially sub-controlled DM (HbA1c 48–59 mmol/mol); and well-controlled DM (HbA1c < 48 mmol/mol). Participants with missing data on HbA1c, self-reported diagnosis of DM and/ self-reported use of anti-diabetic medication were labeled “Missing/ unclassified”.

We merged persons with prediabetes, undiagnosed DM, poorly controlled DM, and potentially sub-controlled DM into one group designated DM-related conditions in need of intervention.

### Body mass index

Body mass index (BMI) was defined as weight in kilograms divided by height in meters squared (kg/m^2^), and divided into underweight (BMI less than 18.5) normal (BMI 18.5–24.9), overweight (BMI 25.0–29.9), and obese (BMI 30.0 or greater) [15].

### Statistical methods

We tabulated the distribution of study participants by DM-related diagnostic groups. Based on the risk factors known from the survey, we calculated relative risks (RR), by unconditional maximum likelihood estimation (MLE), including 95% confidence intervals (CI), using normal approximation, for presence of DM-related conditions in need of intervention. For risks found to be strongly associated with these conditions, we calculated also sensitivity and specificity, and corresponding 95% confidence intervals using exact binomial test, of a hypothesized intervention. Simultaneous confidence intervals for multinomial proportions were calculated with the method of Sison and Glaz (see MultinomialCI package in R and refs. therein). Adjusted RR was obtained with a log-binomial model, and the R package ‘logbin’ was used.

Data handling, statistical analyses and plots were performed in R ver. 3.5.2, using dplyr, epitools, MultinomialCI, VennDiagram and RColorBrewer packages.

## Results

Among the 10,895 LOFUS-participants aged 20 years and above, the prevalence of prediabetes was 5.8% (95% CI: 5.1–6.5). The overall DM prevalence was 6.1% (95% CI: 5.6–6.5) with a diagnosed to undiagnosed DM ratio of 5.3:0.8. The prevalence of undiagnosed DM was 0.8% (95% CI: 0.1–1.4); poorly controlled DM 1.2% (95% CI: 0.5–1.9); potentially sub-controlled DM 2.0% (95% CI: 1.4–2.7); and well-controlled DM 2.1% (95%CI: 1.4–2.8) (Table 1). In total, 9.8% of the participants had DM-related conditions in need of intervention. No DM was found in 81.7% (95% CI: 81.0–82.4) and more frequent in women, 84.3%, than in men, 79.0% (Supplementary Table 1). DM diagnosis was unclassified in 6.4% due to missing values for one or more DM defining factors (Supplementary Figure 1).

**Table 1:** Distribution by diabetes-related diagnostic groups of participants in Lolland-Falster Health Study aged 20 + years: Determinants, 2016–2019

Diagnostic group	Men		Women		Total	
	n	% with (95% CI)	n	% with (95% CI)	n	% with (95% CI)
No DM	4182	79 (78.0–80.1)	4722	84.3 (83.4–85.2)	8904	81.7 (81.0–82.4)
Prediabetes	325	6.1 (5.1–7.2)	308	5.5 (4.6–6.4)	633	5.8 (5.1–6.5)
Undiagnosed DM	55	1.0 (0–2.1)	27	0.5 (0–1.4)	82	0.8 (0.1–1.4)
Poorly controlled DM	81	1.5 (0.5–2.6)	47	0.8 (0–1.7)	128	1.2 (0.5–1.9)
Potentially sub-controlled DM	148	2.8 (1.8–3.9)	75	1.3 (0.4–2.2)	223	2.0 (1.4–2.7)
Well-controlled DM	138	2.6 (1.6–3.7)	90	1.6 (0.7–2.5)	228	2.1 (1.4–2.8)
Missing/ unclassified	362	6.8 (5.8–7.9)	335	6.0 (5.1–6.9)	697	6.4 (5.7–7.1)
Total with missing/ unclassified	5291	100	5604	100	10,895	100
Total excluding missing/ unclassified	4929	93.2	5269	94.0	10,198	93.6
DMRC	609	11.5 (10.7–12.4)	457	8.2 (7.5–8.9)	1066	9.8 (9.2–10.4)
DM diagnosed	367	100	212	100	579	100
- HbA1c ≥ 53 mmol/mol	160	43.6 (38.4–48.9)	88	41.5 (34.9–48.4)	248	42.8 (38.7–47.0)
- HbA1c < 53 mmol/mol	207	56.4 (51.2–61.7)	124	58.5 (51.9–65.4)	331	57.2 (53.0–61.4)

*DMRC* diabetes-related conditions in need of intervention

Among those diagnosed with DM, 42.8% had HbA1c ≥ 53 mmol/mol; a percentage slightly higher for men than for women. In persons diagnosed with DM, 22.1% had poorly controlled DM and 38.5% had potentially sub-controlled DM; percentages increasing with age (Supplementary Table 2).

Mean age was 64.8 years (SD = 10.7) in the prediabetes group, 62.4 years (SD = 10.1) in the undiagnosed DM group, 62.7 years (SD = 11.7) in the poorly controlled DM group, and 66.3 years (SD = 9.3) in the potentially sub-controlled DM group. With regard to lipid profiles, mean HDL concentration was 1.3 ± 0.4 mmol/l in both prediabetes and undiagnosed DM groups, 1.2 ± 0.4 mmol/l in the poorly controlled DM group, and 1.3 ± 0.4 mmol/l in the potentially sub-controlled DM group. Mean total cholesterol concentration was 5.2 ± 1.2 mmol/l in persons with prediabetes; 4.3 ± 1.1 mmol/l in persons with undiagnosed DM and poorly controlled DM, and 4.2 ± 0.9 mmol/l in persons with potentially sub-controlled DM. Mean triglyceride concentration was 2.3 ± 1.4 mmol/l among individuals with prediabetes; 2.2 ± 1.4 mmol/l among individuals with undiagnosed DM, 2.6 ± 2.1 mmol/l among individuals with poorly controlled DM, and 2.2 ± 1.2 mmol/l in individuals with potentially sub-controlled DM (Supplementary Table 3). Definitions of diagnostic groups can be found in Supplementary Table 4.

Men were at a higher risk than women of having a DM-related condition in need of intervention; RR 1.41 (95% CI 1.26–1.58) (Table 2). This RR was almost identical when calculated based on only participants with diagnostic information; RR 1.42 (95% CI 1.27–1.60), reflecting that persons with missing diagnostic information were almost randomly distributed between men and women. In the following, we focus on RRs calculated based only on persons with diagnostic information. Persons aged 60 years and above had a more than doubled risk compared with younger persons; RR 2.66 (95% CI 2.34–3.01). Current and former smoking were associated with slightly increased risks as compared with never smoking; RR of 1.38 (95% CI 1.19–1.62) and 1.45 (95% CI 1.27–1.65), respectively. Self-reported health as being undecided compared with good was associated with an increased risk; RR 1.88 (95% CI 1.67–2.12); and so was poor self-reported health; RR 2.59 (95% CI 2.13–3.15). Presence of self-reported hypertension as compared with absence was associated with a RR of 3.28 (95% CI 2.93–3.68). Low and moderate leisure time physical activity as being compared with high leisure time physical activity; RR of 2.64 (95% CI 2.17–3.22) and 1.84(1.56–2.17). Overweight as compared with normal weight was associated with an increased risk; RR 2.40 (95% CI 2.0–2.87); and so was obesity; RR 4.51 (95% CI 3.79–5.38). The RR for DM-related conditions in obese persons adjusted for health perception, smoking, hypertension, physical activity, and dietary was 3.28 (2.73–3.93).

**Table 2:** Relative Risk of diabetes-related conditions in need of intervention

	Total n (%)	Diagnostic group				RR of DMRC (total)	RR of DMRC excl. Missing/ unclassified	DMRC % excl. Missing/ unclassified
		No DM	DMRC	WCDM	Missing/unclassified			
		n (%)	n (%)	n (%)	n (%)			
Total	10,895 (100)	8904 (81.7)	1066 (9.8)	228 (2.1)	697 (6.4)			10.5
Men	5291 (48.6)	4182 (79.0)	609 (11.5)	138 (2.6)	362 (6.8)	1.41(1.26–1.58)	1.42(1.27–1.60)	12.4
Women	5604 (51.4)	4722 (84.3)	457 (8.2)	90 (1.6)	335 (6.0)	1	1	8.7
Age								
- 20–59	5789 (53.1)	5014 (86.6)	314 (5.4)	36 (0.6)	425 (7.3)	1	1	5.9
- 60+	5106 (46.9)	3890 (76.2)	752 (14.7)	192 (3.8)	272 (5.3)	2.71(2.39–3.08)	2.66(2.34–3.01)	15.6
Smoking								
- Current	1997 (18.3)	1680 (84.1)	228 (11.4)	38 (1.9)	51 (2.6)	1.38(1.19–1.62)	1.39(1.19–1.63)	11.7
- Former	3721 (34.2)	3084 (82.9)	445 (12.0)	118 (3.2)	74 (2.0)	1.45(1.27–1.65)	1.45(1.28–1.65)	12.2
- Never	4658 (42.8)	4116 (88.4)	384 (8.2)	71 (1.5)	87 (1.9)	1	1	8.4
- Missing	519 (4.8)	24 (4.6)	9 (1.7)	1 (0.2)	485 (93.5)	0.21(0.11–0.40)	3.15(1.78–5.56)	26.5
Health status, self-reported								
- Good/very good	7154 (65.7)	6366 (89.0)	559 (7.8)	112 (1.6)	117 (1.6)	1	1	7.9
- Neither good/nor bad	2770 (25.4)	2178 (78.6)	407 (14.7)	100 (3.6)	85 (3.1)	1.88(1.67–2.12)	1.91(1.69–2.15)	15.2
- Poor/very poor	474 (4.4)	342 (72.2)	96 (20.3)	16 (3.4)	20 (4.2)	2.59(2.13–3.15)	2.66(2.19–3.23)	21.2
-Missing/unclassified	497 (4.6)	18 (3.6)	4 (0.8)	0	475 (95.6)	0.10(0.04–0.27)	2.29(0.94–5.57)	18.2
Hypertension, self-reported								
- Yes	3004 (27.6)	2156 (71.8)	606 (20.2)	164 (5.5)	78 (2.6)	3.28(2.93–3.68)	3.33(2.97–3.73)	20.7
- No	7244 (66.5)	6642 (91.7)	445 (6.1)	61 (0.8)	96 (1.3)	1	1	93.0
- Missing/unclassified	647 (5.9)	106 (16.4)	15 (2.3)	3 (0.5)	523 (80.8)	0.38(0.23–0.63)	1.94(1.20–3.15)	12.1
Dietary								
- Very healthy/healthy	4946 (45.4)	4268 (86.3)	473 (9.6)	111 (2.2)	94 (1.9)	1	1	9.8
- Roughly healthy	4901 (45.0)	4155 (84.8)	537 (11.0)	107 (2.2)	102 (2.1)	1.15(1.02–1.29)	1.15(1.02–1.29)	11.2
- Unhealhty/very unhealthy	534 (4.9)	456 (85.4)	50 (9.4)	9 (1.7)	19 (3.6)	0.98(0.74–1.29)	1(0.75–1.31)	9.7
- Missing/unclassified	514 (4.7)	25 (4.9)	6 (1.2)	1 (0.2)	482 (93.8)	0.12(0.05–0.27)	1.92(0.93–3.98)	20.0
Physical activity								
- Low (mainly sedentary)	1245 (11.4)	969 (77.8)	195 (15.7)	47 (3.8)	34 (2.7)	2.64(2.17–3.22)	2.67(2.2–3.26)	16.1
- Moderate (light physical activities ≥ 4 h per week)	6356 (58.3)	5395 (84.9)	694 (10.9)	134 (2.1)	133 (2.1)	1.84(1.56–2.17)	1.85(1.57–2.18)	11.2
- High (sports or other more vigorous activities ≥ 4 h per week/highly vigorous physical activity several times per week)	2732 (25.1)	2484 (91.0)	162 (5.9)	44 (1.6)	42 (1.5)	1	1	6.0
- Missing	562 (5.2)	56 (10.0)	15 (2.7)	3 (0.5)	488 (86.8)	0.45(0.27–0.76)	3.37(2.09–5.42)	20.3
BMI								
- Underweight	124 (1.1)	111 (89.5)	4 (3.2)	0	9 (7.3)	0.80(0.30–2.12)	0.81(0.31–2.16)	3.5
- Normal	3743 (34.4)	3349 (89.5)	151 (4.0)	36 (1.0)	207 (5.5)	1	1	4.3
- Overweight	4260 (39.1)	3470 (81.5)	412 (9.7)	95 (2.2)	283 (6.6)	2.40(2.0–2.87)	2.43(2.02–2.91)	10.4
- Obese	2712 (24.9)	1932 (71.2)	494 (18.2)	95 (3.5)	191 (7.0)	4.51(3.79–5.38)	4.59(3.85–5.46)	19.6
- Missing/unclassified	56 (0.5)	42 (75.0)	5 (9.0)	2 (3.6)	7 (12.5)	2.21(0.94–5.18)	2.39(1.03–5.56)	10.6
Lipids (mean ± SD) *mmol/L*								
HDL	1.5 ± 0.4	1.5 ± 0.4	1.3 ± 0.4	1.3 ± 0.4	1.4 ± 0.4			
Total cholesterol	5.1 ± 1.1	5.2 ± 1.1	4.9 ± 1.2	4.4 ± 1.1	5.0 ± 1.1			
Triglycerides	1.8 ± 1.2	1.7 ± 1.1	2.4 ± 1.5	2.0 ± 1.0	1.9 ± 1.3			

*DMRC* diabetes-related conditions in need of intervention, *WCDM* well-controlled diabetes

Unhealthy dietary habits were not found to be associated with higher risk when compared with healthy/ very healthy; RR of 0.98 (95% CI 0.74–1.29).

The results in Table 2 can form the basis for easy identification in health care and/or social service institutions of persons with DM-related conditions in need of intervention. As 10% of all adult LOFUS-participants had a DM-related condition in need of intervention, we focused on the risk factors showing a doubling of the prevalence proportion. Namely, among persons with poor self-perceived health those with DM-related conditions constituted 21.2%; among persons with self-reported hypertension 20.7%; among persons reporting low leisure time physical activity 16.1%; and among obese persons 19.6% (Table 2).

However, for potential interventions both the sensitivity and the specificity need to be known. Interventions targeting persons with poor self-perceived health would identify only 9.0% (95% CI 7.4–10.9) of persons with DM-related conditions in need of intervention, but it would on the other hand cause limited harm as it would target very few persons without the conditions; the specificity being 96.1% (95% CI 95.7–96.5) (Table 3).

**Table 3:** Sensitivity and specificity of detecting diabetes-related conditions in need of intervention

Exposure	Exposed		Non-exposed		Sensitivity (95% CI)	Specificity (95% CI)	Positive predictive value (95% CI)	Negative predictive value (95% CI)
	DMRC n	Non-DMRC n	DMRC n	Non-DMRC n				
Age 60+	752	4082	314	5050	70.5%(67.7–73.3)	55.3%(54.3–56.3)	15.6%(14.5–16.6)	94.1%(93.5–94.8)
Poor/very poor health status, self-reported	96	358	966	8756	9.0%(7.4–10.9)	96.1%(95.7–96.5)	21.1%(17.5–25.2)	90.1%(89.5–90.7)
Hypertension, self-reported	606	2320	445	6703	57.7%(54.6–60.7)	74.3%(73.4–75.2)	20.7%(19.3–22.2)	93.8%(93.2–94.3)
Low physical activity	195	1016	856	8057	18.3%(16.2–21.0)	88.8%(88.1–89.4)	16.1%(14.1–18.3)	90.4%(89.8–91.0)
Obesity, measured	494	2027	567	7061	46.6%(43.5–49.6)	77.7%(76.8–78.5)	19.6%(18.1–21.2)	92.6%(92.0–93.1)

*DMRC* diabetes-related conditions in need of intervention

Interventions targeting persons with low leisure time physical activity would identify 18.3% (95% CI 16.2–21.0) of persons with DM-related conditions in need of intervention, with a specificity of 88.8 (CI 88.1–89.4). Intervention towards obese persons or persons with hypertension would identify between 46.6% (95% CI 43.5–49.6) and 57.7% (95% CI 54.6–60.7) of the persons with the DM-related conditions in need of intervention, and with specificities between 74.3% (95% CI 73.4–75.2) and 77.7% (95% CI 76.8–78.5) only about one fourth of targeted persons would be without the conditions.

For comparison, we calculated also sensitivity and specificity for an intervention towards all persons aged 60 years and above. An intervention towards this group would be effective, as 70.5% (95% CI 67.7–73.3) of those with the DM-related conditions would be identified, but with a specificity of only 55.3% (95% CI 54.3–56.3) almost half of the targeted persons would not have the conditions.

Cardiovascular disease was reported in 55.3% of adults with a DM-related condition in need of intervention, and cardiovascular medication in 68.9%; 24.7 and 29.7% in adults with no DM; 72.0 and 85.1% in persons with well-controlled DM. Micro- and macro-albuminuria were observed more frequently in persons with DM-related conditions in need of intervention, 22.1 and 4.0%, respectively, than in persons with no DM, 11.6 and 0.7%, respectively (Table 4).

**Table 4:** Cardiovascular disease and albuminuria

	Total n (%)	No DM n (%)	DMRC n (%)	WCDM n (%)	Missing/ unclassified n (%)
Overall	10,895 (100)	8904 (81.7)	1066 (9.8)	228 (2.1)	697 (6.4)
Cardiovascular medication self-reported^a^					
- Yes	3666 (33.7)	2643 (29.7)	734 (68.9)	194 (85.1)	95 (13.6)
- No	6580 (60.4)	6145 (69.0)	317 (29.7)	33 (14.5)	85 (12.2)
- Missing/ unclassified	649 (6.0)	116 (1.3)	15 (1.4)	1 (0.4)	517 (74.2)
Cardiovascular disease self-reported^a^					
- Yes	3016 (27.7)	2197 (24.7)	589 (55.3)	164 (72.0)	66 (9.5)
- No	7400 (67.9)	6707 (75.3)	477 (44.7)	64 (28.1)	152 (21.8)
- Missing/unclassified	479 (4.4)	0	0	0	479 (68.7)
Urine albumin-to-creatinine ratio mg/g					
- 0–29	6106 (56.0)	5047 (56.7)	567 (53.2)	106 (46.5)	386 (55.4)
- 30–299 (micro albuminuria)	1410 (13.0)	1034 (11.6)	236 (22.1)	51 (22.4)	89 (12.8)
- ≥ 300 (macro albuminuria)	130 (1.2)	66 (0.7)	43 (4.0)	12 (5.3)	9 (1.3)
- Missing/unclassified	3249 (29.8)	2757 (31.0)	220 (20.6)	59 (25.9)	213 (30.6)

*DMRC* diabetes-related conditions in need of intervention, *WCDM* well-controlled diabetes

^a^Cardiovascular disease and medication includes hypertension diagnosis and antihypertensive medication

## Discussion

### Main findings

One in 10 participants in the rural-provincial Lolland-Falster Health Study had a DM-related condition that required further investigation and/or better glycaemic control. As these conditions may lead to serious health consequences, the study revealed a considerable unmet need for prevention. The risk of undiagnosed and/or poorly controlled conditions was more than doubled among persons with self-reported poor health status; with self-reported hypertension; with low self-reported leisure time physical activities or with obesity. Within these risk groups, 20% had an undiagnosed and/or poorly controlled condition. On this basis, health care and social service workers could in general recommend persons with these characteristics to have a blood sample analyzed and to seek advice on treatment. Provided that these services are easily available, such a simple, targeted strategy could potentially save many persons from developing severe life-long complications of DM.

### Comparison with other studies

The prevalence of prediabetes of 5.8% in the LOFUS population was slightly higher than the 4.3% found in the Danish Health Examination Survey (DANHES), but lower than the 6.9% in the Danish General Suburban Population Study (GESUS) [6].

The prevalence of undiagnosed and diagnosed DM was 0.8 and 5.3%, respectively. The prevalence was higher in men than in women, and while the reasons for this remain poorly understood, biological and psychosocial factors may play a role [16, 17], and women visit primary care clinics and diagnostic services more frequently than men [18, 19]. Our prevalence of undiagnosed DM of 0.8% was higher than the 0.3% in GESUS and the 0.2% in the urban Copenhagen General Population Study (CGPS) [20], but lower than the 1.4% in DANHES based on slightly different definitions and data sources [6].

Our findings on health related characteristics associated with prediabetes and DM were consistent with previous studies; low leisure time physical activity and obesity are well established risk factors for DM-related conditions [21, 22] and so is hypertension as DM and high blood pressure share a number of potential risk factors, and are likely to cluster together along with other cardio-metabolic conditions [23]. The association between poor self-reported health status and DM-related conditions collaborate findings from Italy of a high correlation between elevated HbA1c concentrations and poor self-reported health status [24], and from Sweden where adults with high-risk HbA1c concentrations reported poor health status and had low level of health related quality of life [25]. Unhealthy dietary habits are associated with metabolic changes and increased risk of DM-related conditions [26]. However, this was not found in our study and could be related to the less comprehensive questionnaire applied.

The risk of DM increases with age resulting from increasing insulin resistance and impaired pancreatic islet function [27]. Older adults with DM are also at substantial risk for both acute and chronic macrovascular and cardiovascular complications. Our findings on age as a risk factor is therefore in concordance with the literature [28].

Triglyceride concentrations were slightly higher in the prediabetes, undiagnosed DM and poorly controlled DM groups when compared with the no DM group, consistent with previous studies [28, 29]. Some previous studies have reported lower HDL levels and higher total cholesterol concentrations in persons with prediabetes and DM [23, 30], but this was not found in our population.

Adults with DM-related conditions in need of intervention reported more frequently having cardiovascular disease or using cardiovascular medication than adults with no DM. There is evidence that chronic high blood glucose levels are associated with a substantially higher risk of cardiovascular disease [31], and patients with cardiovascular disease are reported to have a high risk of developing DM [32]. Because of the known relationship between cardiovascular disease and DM, early detection of DM related conditions in need of intervention are essential in order to prevent and delay progressive conditions.

A substantial proportion of adults with a DM-related condition in need of intervention had evidence of micro- and macro-albuminuria; identification and follow up is necessary to prevent and delay chronic kidney damage in this group. Previous studies found it might be efficient to test kidney function in adults with prediabetes or in persons at risk of having DM in order to determine chronic kidney disease at an early stage to prevent further complications [33, 34].

In LOFUS, approximately 43% of the persons diagnosed with DM had HbA1c levels ≥ 53 mmol/mol. Similar percentages have been reported in other countries globally [35, 36]. In the U.S. National Health and Nutrition Examination Survey (NHANES) from 2007 to 2010, 47.5% of adults with DM had HbA1c levels ≥ 53 [35]. In a Korean survey, 50.9% of adults with DM had HbA1c ≥ 53 in 2010 [36]. Although these studies differed in population, sample sizes, and survey period, the results showed that glycemic control is challenged around the globe.

### Limitations

This cross-sectional study had a number of limitations. First, only 34.1% of persons invited participated in LOFUS, which may lead to selection bias. There was an overall higher participation rate among individuals of higher income, education level, employment status, middle-aged (aged 50–60 years) and married [10]. It is already known that survey participation is lower among people of poorer socio-economic status and with disease compared with those without [37]. This can result in underestimation of the prevalence of prediabetes, undiagnosed DM, and poorly controlled DM, if the survey prevalence is taken at face value. Thus, our results likely represented conservative estimates of the true unmet need in a deprived area of Denmark. Second, while we were not able to distinguish between T1DM and T2DM, we assumed that only a small number of participants had T1DM. Third, our definitions of undiagnosed DM, prediabetes and poorly controlled DM may cause underestimation of conditions due to missing data, see Supplementary Figure 1. Fourth, data on alcohol consumption were considered unreliable and not used in the analysis. Moreover, oral glucose tolerance test (OGTT) was not performed in LOFUS and therefore only HbA1c measurements were used for the analyses. Finally, differences in methods for the analysis of HbA1c across surveys influenced the comparison of estimates; however, it is not possible to determine the direction of such potential bias.

### Clinical implications

Long-term care of DM patients is difficult and presents a considerable challenge to health care systems [38]. In order to evaluate DM management, it has been widely agreed that glycaemic control along with lipid profiles and blood pressure is the key in preventing complications. According to the American Diabetes Association, a reasonable target HbA1c value for many non-pregnant adults DM patients is 53 mmol/mol [12]. However, it has been suggested that an individualized therapeutic approach rather than a ‘one-size-fits-all’ strategy is essential to ensuring maximum success [14, 38].

Several DM risk scoring tools, including the Danish Diabetes Risk Score and the Leicester Risk Assessment Score, are available in order to enhance detection of undiagnosed DM [39, 40]. These scores are based on simple anamnestic information and clinical observations and are recommended since uncertainty persists concerning benefits of population-based screening for DM [41]. However, these tools are primarily applied by medical doctors and used for consultations regarding DM.

Our study indicates that a substantial part of persons with DM-related conditions that would require further diagnostics and/or improved glycaemic control could be identified based on self-reported poor health and/or hypertension, and/ or low leisure time physical activity, and/or presence of obesity. This information is easily available for health care and social service workers simply from asking the person and from visually evaluating their body constitution. On this basis, health care and social service workers could in general recommend citizens with these characteristics to have a blood sample analyzed and to seek advice on treatment. Provided that these services are easily available for the citizens, such a simple, targeted strategy could potentially save many persons from developing severe life-long complications of DM.

## Conclusion

In the Lolland-Falster Health Study, about 10% of participants had prediabetes, undiagnosed DM, poorly controlled or potentially sub-controlled DM. The risk of these conditions was more than doubled in persons with poor self-perceived health, self-reported hypertension, low leisure time physical activity or obesity. These findings can form the basis for a targeted effort to improve the prevention of DM and its complications.

## Supplementary Information

**Additional file 1 **: **Supplementary Table 1**. Prevalence of prediabetes, undiagnosed and diagnosed diabetes according to gender and age. **Supplementary Table 2**. Diagnosed diabetes disaggregated by glycemic control according to gender and age. **Supplementary Table 3**. Descriptive characteristics of LOFUS participants by glycemic status. **Supplementary Table 4**. Definitions of diagnostic groups. **Supplementary Figure 1**. Venn diagram for missing/ unclassified data for one or more diabetes defining factors.

## Data Availability

Data from the study can be made available via Region Sjaelland following the Danish Data Protection Regulation.

## Abbreviations

DM: Diabetes mellitus
IDF: The International Diabetes Federation
T1DM: Type 1 DM
T2DM: Type 2 DM
LOFUS: The Lolland-Falster Health Study
HbA1c: Glycated hemoglobin
HDL: High-density lipoprotein cholesterol
UACR: Urine albumin-to-creatinine ratio
ADA: The American Diabetes Association
BMI: Body mass index
RR: Relative risks
MLE: Maximum likelihood estimation
CI: Confidence intervals
DANHES: The Danish Health Examination Survey
GESUS: The Danish General Suburban Population Study
CGPS: The Copenhagen General Population Study
OGTT: Oral glucose tolerance test
NHANES: National Health and Nutrition Examination Survey
DMRC: Diabetes-related conditions in need of intervention
PCDM: Poorly controlled diabetes
PSCDM: Potentially sub-controlled diabetes
WCDM: Well-controlled diabetes

## References

[1] American Diabetes Association (2009). Diagnosis and classification of diabetes mellitus. Diabetes Care.

[2] Cho NH, Shaw JE, Karuranga S (2018). IDF diabetes atlas: global estimates of diabetes prevalence for 2017 and projections for 2045. Diabetes Res Clin Pract.

[3] Valdés S, Botas P, Delgado E (2009). CadórnigaFD. Mortality risk in Spanish adults with diagnosed diabetes, undiagnosed diabetes, or pre-diabetes. The Asturias study 1998–2004. Re Esp Cardiol.

[4] Wild SH, Smith FB, Lee AJ, Fowkes FG (2005). Criteria for previously undiagnosed diabetes and risk of mortality: 15- year follow-up of the Edinburgh artery study cohort. Diabet Med.

[5] Carstensen B, Kristensen JK, Ottosen P, Borch-Johnsen K, Steering Group of the National Diabetes Register (2008). The Danish National Diabetes Register: trends in incidence, prevalence and mortality. Diabetologia.

[6] Jørgensen ME, Ellervik C, Ekholm O, Johansen NB, Carstensen B. Estimates of prediabetes and undiagnosed type 2 diabetes in Denmark: the end of an epidemic or a diagnostic arte- fact? Scand J Public Health. 2018:1–7. 10.1177/1403494818799606 30222048

[7] Statistics Denmark: https://www.statistikbank.dk/10083. [Accessed 6 January 2020 (data reference item accessed on organization website)].

[8] Blaakilde AL, Hansen BH, Olesen LS (2018). Health profile 2017 for region Zealand and municipalities - “How are you?”(in Danish).

[9] Jepsen R, Egholm CL, Brodersen J, et al. Lolland-Falster health study: study protocol for a household-based prospective cohort study. Scand J Public Health. 2020;48:382–90. 10.1177/1403494818799613 PMC7263040 30222051

[10] Jepsen R, Wingstrand A, Abild SL, et al. Socio-economic determinants of participation in the Lolland-Falster health study. J Public Health (Berl.). 2019. 10.1007/s10389-019-01095-z.

[11] Bowling A. Just one question: if one question works, why ask several? J Epidemiol Community Health. 2005;59:342–5. 10.1136/jech.2004.021204 PMC1733095 15831678

[12] Danmarks Statistik: https://www.statistikbank.dk/10083 [Accessed 6 January 2020 (data reference item accessed on organization website)].

[13] Dansk Selskab for Almen Medicin, Danish Association for General Practice, Clinical Guideline. Type 2 Diabetes - Et metabolisk syndrom. Copenhagen, Denmark: http://vejledninger.dsam.dk/type2 [Accessed 17 December 2019 (data reference item accessed on organization website)].

[14] Standards of Medical Care in Diabetes—2019 Abridged for Primary Care Providers: American Diabetes Association Clinical Diabetes 2019; 37:11–34. 10.2337/cd18-0105 PMC6336119 30705493

[15] World Health Organization. In: WHO, editor. Obesity: Preventing and Managing the Global Epidemic. Report of a WHO Consultation on Obesity. Geneva; 1998. Geneva, 3–5 June 1997. WHO Technical Report Series 894. 11234459

[16] Kautzky-Willer A, Harreiter J, Pacini G (2016). Sex and gender differences in risk, pathophysiology and complications of type 2 diabetes mellitus. Endocr Rev.

[17] DECODE Study Group (2003). Age- and sex-specific prevalences of diabetes and impaired glucose regulation in 13 European cohorts. Diabetes Care.

[18] Bertakis KD, Azari R, Helms LJ, Callahan EJ, Robbins JA (2000). Gender differences in the utilization of health care services. J Fam Pract.

[19] Manandhar M, Hawkes S, Buse K, Nosrati E, Magar V (2018). Gender, health and the 2030 agenda for sustainable development. Bull World Health Organ.

[20] Bergholdt HK, Bathum L, Kvetny J (2013). Study design, participation and characteristics of the Danish general suburban population study. Dan Med J.

[21] Boucher AB, Adesanya EA, Owei I (2015). Dietary habits and leisure-time physical activity in relation to adiposity, dyslipidemia, and incident Dysglycemia in the pathobiology of Prediabetes in a biracial cohort study. Metabolism.

[22] Meisinger C, Löwel H, Thorand B, Döring A (2005). Leisure time physical activity and the risk of type 2 diabetes in men and women from the general population. The MONICA/KORA Augsburg Cohort Study. Diabetologia.

[23] Qiu M, Shen W, Song X (2015). Effects of prediabetes mellitus alone or plus hypertension on subsequent occurrence of cardiovascular disease and diabetes mellitus: longitudinal study. Hypertension.

[24] Nicolucci A, Cucinotta D, Squatrito S (2009). Clinical and socio-economic correlates of quality of life and treatment satisfaction in patients with type 2 diabetes. Nutr Metab Cardiovasc Dis.

[25] Engström MS, Leksell J, Johansson UB (2019). Health-related quality of life and glycaemic control among adults with type 1 and type 2 diabetes – a nationwide cross-sectional study. Health Qual Life Outcomes.

[26] Sami W, Ansari T, Butt NS, Hamid MRA (2017). Effect of diet on type 2 diabetes mellitus: a review. Int J Health Sci.

[27] Chang AM, Halter JBA (2003). Aging and insulin secrection. J Physiol Endocrinol Metabol.

[28] Wu Y, Ding Y, Tanaka Y, Zhang W (2014). Risk factors contributing to type 2 diabetes and recent advances in the treatment and prevention. Int J Med Sci.

[29] Zhu ZW, Denga FY, Lei SF (2015). Meta-analysis of Atherogenic index of plasma and other lipid parameters in relation to risk of type 2 diabetes mellitus. Prim Care Diabetes.

[30] Stöckl D, Rückert-Eheberg IM, Heier M (2016). Regional variability of lifestyle factors and hypertension with prediabetes and newly diagnosed type 2 diabetes mellitus: the population-based kora-f4 and ship-trend studies in Germany. PLoS One.

[31] Nielson C, Lange T (2005). Blood glucose and heart failure in nondiabetic patients. Diabetes Care.

[32] Tenenbaum A, Fisman EZ (2004). Impaired glucose metabolism in patients with heart failure: pathophysiology and possible treatment strategies. Am J Cardiovasc Drugs.

[33] Vassalotti JA, Li S, Chen SC, Collins AJ. Screening populations at increased risk of CKD: the kidney early evaluation program (KEEP) and the public health problem. Am J Kidney Dis. 2009:107–14. 10.1053/j.ajkd.2008.07.049 19231754

[34] Plantinga LC, Crews DC, Coresh J, et.al. Prevalence of chronic kidney disease in US adults with undiagnosed diabetes or prediabetes. Clin J Am Soc Nephrol 2010; 673–682. 10.2215/CJN.07891109 PMC2849697 20338960

[35] Stark Casagrande S, Fradkin JE, Saydah SH, Rust KF, Cowie CC (2013). The prevalence of meeting A1C, blood pressure, and LDL goals among people with diabetes, 1988–2010. Diabetes Care.

[36] Yu SH, Kang JG, Hwang YC (2013). Increasing achievement of the target goals for glycemic, blood pressure and lipid control for adults with diagnosed diabetes in Korea. J Diabetes Invest.

[37] Larsen SB, Dalton SO, Schüz J (2012). Mortality among participants and non-participants in a prospective cohort study. Eur J Epidemiol.

[38] Blonde L, Aschner P, Bailey C (2017). Gaps and barriers in the control of blood glucose in people with type 2 diabetes. Diab Vasc Dis Res.

[39] Glumer C, Carstensen B, Sandbaek A (2004). Inter99 study A Danish diabetes risk score for targeted screening: the Inter99 study. Diabetes Care.

[40] Gray LJ, Taub NA, Khunti K (2010). The Leicester risk assessment score for detecting undiagnosed type 2 diabetes and impaired glucose regulation for use in a multiethnic UK setting. Diabet Med.

[41] Lau CJ, Pisinger C, Husemoen LL (2016). Effect of general health screening and lifestyle counselling on incidence of diabetes in general population: Inter99 randomised trial. Prev Med.

[42] Sundhed.dk, eHealth in Denmark: https://www.sundhed.dk/borger/service/om-sundheddk/ehealth-in-denmark/ [Accessed 17 December 2019 (data reference item accessed on organization website)].

