# Supplementary material for: Burden of prediabetes, undiagnosed, and poorly or potentially sub-controlled diabetes: Lolland-Falster health study
Source: BMC Public Health. 2020 Nov 16;20:1711. doi: 10.1186/s12889-020-09791-2 (PMC7667788; doi:10.1186/s12889-020-09791-2)

**Supplementary Table 1. Prevalence of prediabetes, undiagnosed and diagnosed diabetes according to gender and age**

|  | Total  n | No DM  n (%) | Prediabetes  n (%) | Undiagnosed DM  n (%) | Diagnosed DM  n (%) | Missing/ unclassified  n (%) |
| --- | --- | --- | --- | --- | --- | --- |
| Overall | 10895 | 8904 (81.7) | 633 (5.8) | 82 (0.8) | 579 (5.3) | 697 (6.4) |
| Males (years) | 5291 | 4182 (79.0) | 325 (6.1) | 55 (1.0) | 367 (7.1) | 362 (6.9) |
| - 20-29 | 340 | 295 (86.8) | 0 | 1 (0.3) | 2 (0.5) | 42 (12.4) |
| - 30-39 | 420 | 366 (87.1) | 7 (1.7) | 0 | 0 | 47 (11.2) |
| - 40-49 | 743 | 640 (86.1) | 13 (1.7) | 4 (0.5) | 17 (2.4) | 69 (9.3) |
| - 50-59 | 1151 | 946 (82.2) | 69 (6.0) | 17 (1.5) | 55 (4.7) | 64 (5.6) |
| - 60-69 | 1363 | 1022 (75.0) | 120 (8.8) | 17 (1.2) | 146 (10.7) | 58 (4.3) |
| - 70-79 | 1005 | 733 (72.9) | 82 (8.2) | 14 (1.4) | 122 (12.1) | 54 (5.4) |
| - >80 | 269 | 180 (67.0) | 34 (12.6) | 2 (0.7) | 25 (9.9) | 28 (10.4) |
| Females (years) | 5604 | 4722 (84.3) | 308 (5.5) | 27 (0.5) | 212(3.7) | 335(6.0) |
| - 20-29 | 343 | 303 (88.3) | 1 (0.3) | 0 | 1(0.3) | 38(11.1) |
| - 30-39 | 490 | 442 (90.2) | 4 (0.8) | 0 | 1(0.2) | 43(8.8) |
| - 40-49 | 929 | 828 (89.1) | 24 (2.6) | 2 (0.2) | 17(1.9) | 58(6.2) |
| - 50-59 | 1373 | 1194 (87.0) | 67 (4.9) | 9 (0.7) | 39(3.0) | 64(4.7) |
| - 60-69 | 1367 | 1125 (82.3) | 116 (8.5) | 11 (0.8) | 73(5.3) | 42(3.1) |
| - 70-79 | 897 | 683 (76.1) | 81 (9.0) | 4 (0.5) | 67(7.6) | 62(6.9) |
| - >80 | 205 | 147 (71.7) | 15 (7.3) | 1 (0.5) | 14(6.8) | 28(13.7) |

**Supplementary Table 2. Diagnosed diabetes disaggregated by glycemic control according to gender and age**

|  | Total  n | HbA1c ~~>~~≥53 mmol/mol  n (%) | HbA1c <53 mmol/mol  n (%) | PCDM^A^  n (%) | PSCDM^B^  n (%) | WCDM^C^  n (%) |
| --- | --- | --- | --- | --- | --- | --- |
| Overall | 579 | 248 (42.8) | 331 (57.2) | 128 (22.1) | 223 (38.5) | 228 (39.4) |
| Males (years) | 367 | 160 (43.6) | 207 (56.4) | 81 (22.1) | 148 (40.3) | 138 (37.6) |
| - 20-29 | 2 | 2 (100) | 0 | 2 (100) | 0 | 0 |
| - 30-39 | 0 | 0 | 0 | 0 | 0 | 0 |
| - 40-49 | 17 | 8 (47.1) | 9 (53.0) | 6 (35.3) | 6 (35.3) | 5 (29.4) |
| - 50-59 | 55 | 26 (47.3) | 29 (52.7) | 15 (27.3) | 26 (47.3) | 14 (25.5) |
| - 60-69 | 146 | 66 (45.2) | 80 (54.8) | 31 (21.2) | 60 (41.1) | 55 (37.7) |
| - 70-79 | 122 | 53 (43.4) | 69 (56.6) | 24 (19.7) | 50 (41.0) | 48 (39.3) |
| - >80 | 25 | 5 (20.0) | 20 (80.0) | 3 (12.0) | 6 (24.0) | 16 (64.0) |
| Females (years) | 212 | 88 (41.5) | 124 (58.5) | 47 (22.2) | 75 (35.4) | 90 (42.5) |
| - 20-29 | 1 | 1 (100) | 0 | 1 (100) | 0 | 0 |
| - 30-39 | 1 | 1 (100) | 0 | 1 (100) | 0 | 0 |
| - 40-49 | 17 | 8 (47.1) | 9 (53.0) | 5 (29.4) | 5 (29.4) | 7 (41.2) |
| - 50-59 | 39 | 22 (56.4) | 17 (43.6) | 14 (35.9) | 15 (38.5) | 10 (25.6) |
| - 60-69 | 73 | 28 (38.4) | 45 (61.6) | 14 (19.2) | 22 (30.1) | 37 (50.7) |
| - 70-79 | 67 | 23 (34.3) | 33 (65.7) | 10 (15.0) | 28 (41.8) | 29 (43.3) |
| - >80 | 14 | 5 (35.7) | 9 (64.3) | 2 (14.3) | 5 (35.7) | 7 (50) |

PCDM^A^ = poorly controlled diabetes, PSCDM^B^ = potentially sub-controlled diabetes, WCDM^C^ = well-controlled diabetes

**Supplementary Table 3. Descriptive characteristics of LOFUS participants by glycemic status**

|  | Total  n(%) | No DM  n(%) | Prediabetes  n(%) | | Undiagnosed  DM n(%) | | Diagnosed  DM n(%) | Missing/ un-unclassified n(%) | PCDM^A^  n(%) | PSCDM^B^  n(%) | WCDM^C^  n(%) | Total  n(%) |
| --- | --- | --- | --- | --- | --- | --- | --- | --- | --- | --- | --- | --- |
| Total | 10895(100) | 8904(81.7) | | 633(5.8) | | 82(0.8) | 579(5.3) | 697(6.4) | 128(22.1) | 223(38.5) | 228(39.4) | 579(100) |
| Age (mean ±SD) | 56.6 ± 15.2 | 55.6 ± 15.1 | | 64.8 ± 10.7 | | 62.4± 10.1 | 66±10.2 |  | 62.7±11.7 | 66.3±9.3 | 67.5±9.9 |  |
| Sex |  |  | |  | |  |  |  |  |  |  |  |
| Male | 5291(48.6) | 4182(79.0) | | 325(6.1) | | 55(1.0) | 367(7.0) | 362(6.8) | 81(22.1) | 148(40.3) | 138(37.6) | 367(63.4) |
| Female | 5604(51.4) | 4722(84.3) | | 308(5.5) | | 27(0.5) | 212(3.8) | 335(6.0) | 47(22.2) | 75(35.4) | 90(42.5) | 212(36.6) |
| Smoking |  |  | |  | |  |  |  |  |  |  |  |
| - Current | 1997(18.3) | 1680(84.1) | | 140(7.0) | | 18(0.9) | 108(5.4) | 51(2.6) | 27(25.0) | 43(39.8) | 38(35.2) | 108(18.7) |
| - Former | 3721(34.2) | 3084(82.9) | | 260(7.0) | | 33(0.9) | 270(7.3) | 74(2.0) | 57(21.1) | 95(35.2) | 118(43.7) | 270(46.6) |
| - Never | 4658(42.8) | 4116(88.4) | | 225(4.8) | | 31(0.7) | 199(4.3) | 87(1.9) | 44(22.1) | 84(42.2) | 71(35.7) | 199(34.4) |
| - Missing/unclassified | 519(4.8) | 24(4.6) | | 8(1.5) | | 0 | 2(0.4) | 485(93.5) | 0 | 1(50) | 1(50) | 2(0.3) |
| Health status,  self-reported |  |  | |  | |  |  |  |  |  |  |  |
| - Good/very good | 7154(65.7) | 6366(89.0) | | 369(5.2) | | 46(0.6) | 256(3.6) | 117(1.6) | 43(16.8) | 101(39.5) | 112(43.8) | 256(44.2) |
| - Neither good/nor bad | 2770(25.4) | 2178(78.6) | | 213(7.7) | | 29(1.1) | 265(9.6) | 85(3.1) | 68(53.1) | 97(43.5) | 100(43.9) | 265(45.8) |
| - Poor/very poor | 474(4.4) | 342(72.2) | | 48(10.1) | | 7(1.5) | 57(12.0) | 20(4.2) | 17(13.3) | 24(10.8) | 16(7.1) | 57(9.8) |
| - Missing/unclassified | 497(4.6) | 18(3.6) | | 3(0.6) | | 0 | 1(0.2) | 475(95.6) | 0 | 1(0.4) | 0 | 1(0.2) |
| Hypertension,  self-reported |  |  | |  | |  |  |  |  |  |  |  |
| - Yes | 3004(27.6) | 2156(71.8) | | 293(9.8) | | 43(1.4) | 434(14.5) | 78(2.6) | 89(20.5) | 181(41.7) | 164(37.8) | 434(75.0) |
| - No | 7244(66.5) | 6642(91.7) | | 330(4.6) | | 39(0.5) | 137(1.9) | 96(1.3) | 36(26.3) | 40(29.2) | 61(44.5) | 137(23.7) |
| - Missing/unclassified | 647(5.9) | 106(16.4) | | 10(1.6) | | 0 | 8(1.2) | 523(80.8) | 3(37.5) | 2(25.0) | 3(37.5) | 8(1.4) |
| Dietary |  |  | |  | |  |  |  |  |  |  |  |
| - Very healthy/healthy | 4946(45.4) | 4268(86.3) | | 286(5.8) | | 29(0.6) | 269(5.4) | 94(1.9) | 54(20.1) | 104(38.7) | 111(41.3) | 269(46.5) |
| - Roughly healthy | 4901(45.0) | 4155(84.8) | | 315(6.4) | | 44(0.9) | 285(5.8) | 102(2.1) | 65(22.8) | 113(39.6) | 107(37.5) | 285(49.2) |
| - Unhealthy/very unhealthy | 534(4.9) | 456(85.4) | | 29(5.4) | | 9(1.7) | 21(4.0) | 19(3.6) | 8(38.1) | 4(19.0) | 9(42.9) | 21(3.6) |
| - Missing/unclassified | 514(4.7) | 25(4.9) | | 3(0.6) | | 0 | 4(0.8) | 482(93.8) | 1(25.0) | 2(50.0) | 1(25.0) | 4(0.7) |
| Physical activity |  |  | |  | |  |  |  |  |  |  |  |
| - Low (mainly sedentary) | 1245(11.4) | 969(77.8) | | 105(8.4) | | 16(1.3) | 121(9.7) | 34(2.7) | 28(23.1) | 46(38.0) | 47(38.8) | 121(20.9) |
| - Moderate (light physical activities >4 hours per week) | 6356(58.3) | 5395(84.9) | | 430(6.8) | | 55(0.9) | 343(5.4) | 133(2.1) | 75(21.9) | 134(39.1) | 134(39.1) | 343(59.2) |
| - High (sports or other more vigorous activities >4 hours per week)/highly vigorous physical activity several times per week | 2732(25.1) | 2484(90.9) | | 89(3.3) | | 10(0.4) | 107(3.9) | 42(1.5) | 25(23.4) | 38(35.5) | 44(41.1) | 107(18.5) |
| - Missing | 562 (5.2) | 56(10.0) | | 9(1.6) | | 1(0.2) | 8(1.42) | 488 (86.8) | 0 | 5(62.5) | 3 (37.5) | 8(1.4) |
| BMI, measured |  |  | |  | |  |  |  |  |  |  |  |
| - Underweight | 124(1.1) | 111(89.5) | | 3(2.4) | | 0 | 1(0.8) | 9(7.3) | 0 | 1(100) | 0 | 1(0.2) |
| - Normal | 3743(34.4) | 3349(89.5) | | 104(2.8) | | 7(0.2) | 76(2.0) | 207(5.5) | 18(23.7) | 22(29.0) | 36(47.4) | 76(13.1) |
| - Overweight | 4260(39.1) | 3470(81.5) | | 250(5.9) | | 27(0.6) | 230(5.4) | 283(6.6) | 51(22.2) | 84(36.5) | 95(41.4) | 230(39.7) |
| - Obese | 2712(24.9) | 1932(71.2) | | 273(10.1) | | 47(1.7) | 269(10.0) | 191(7.0) | 58(21.6) | 116(43.1) | 95(35.3) | 269(46.5) |
| - Missing/ unclassified | 56(0.5) | 42(75.0) | | 3(5.4) | | 1(1.8) | 3(5.4) | 7(12.5) | 1(33.3) | 0 | 2(66.7) | 3(0.5) |
| Lipids (mean ±SD)  mmol/L |  |  | |  | |  |  |  |  |  |  |  |
| HDL | 1.5 ± 0.4 | 1.5 ± 0.4 | | 1.3 ± 0.4 | | 1.3±0.4 | 1.1±0.3 | 1.4 ± 0.4 | 1.2±0.4 | 1.3±0.4 | 1.3±0.4 |  |
| Total cholesterol | 5.1 ± 1.1 | 5.2 ± 1.1 | | 5.2 ± 1.2 | | 4.3±1.1 | 5.2±1.2 | 5.0 ± 1.1 | 4.3±1.1 | 4.2±0.9 | 4.4±1.1 |  |
| Triglycerides | 1.8 ± 1.2 | 1.7 ± 1.1 | | 2.3 ± 1.4 | | 2.2±1.4 | 2.7±1.4 | 1.9 ± 1.3 | 2.6±2.1 | 2.2±1.2 | 2.0±1.0 |  |

PCDM^A^= poorly controlled diabetes, PSCDM^B^ = potentially sub-controlled diabetes, WCDM^C^ = well-controlled diabetes

**Supplementary Table 4. Definitions of diagnostic groups**

| Diagnostic group | HbA1c (mmol/mol) | Antidiabetic medication Insulin (Yes/No/NA) | Antidiabetic medication Other (Yes/No/NA) | Diabetes diagnosis (Yes/No/NA) |
| --- | --- | --- | --- | --- |
| No DM | HbA1c < 42 | No | No | No |
| Prediabetes | 42 < HbA1c < 47 | No | No | No |
| Undiagnosed DM | HbA1c > 48 | No | No | No |
| Diagnosed DM | Any level | At least one Yes |  |  |
| Poorly controlled DM | HbA1c > 60 | At least one Yes |  |  |
| Potentially sub- controlled DM | 48 < HbA1c < 59 | At least one Yes |  |  |
| Well-controlled DM | HbA1c < 48 | At least one Yes |  |  |
| Missing/unclassified | Missing | Any answer |  |  |

NA = not available

**Supplementary Figure 1.**


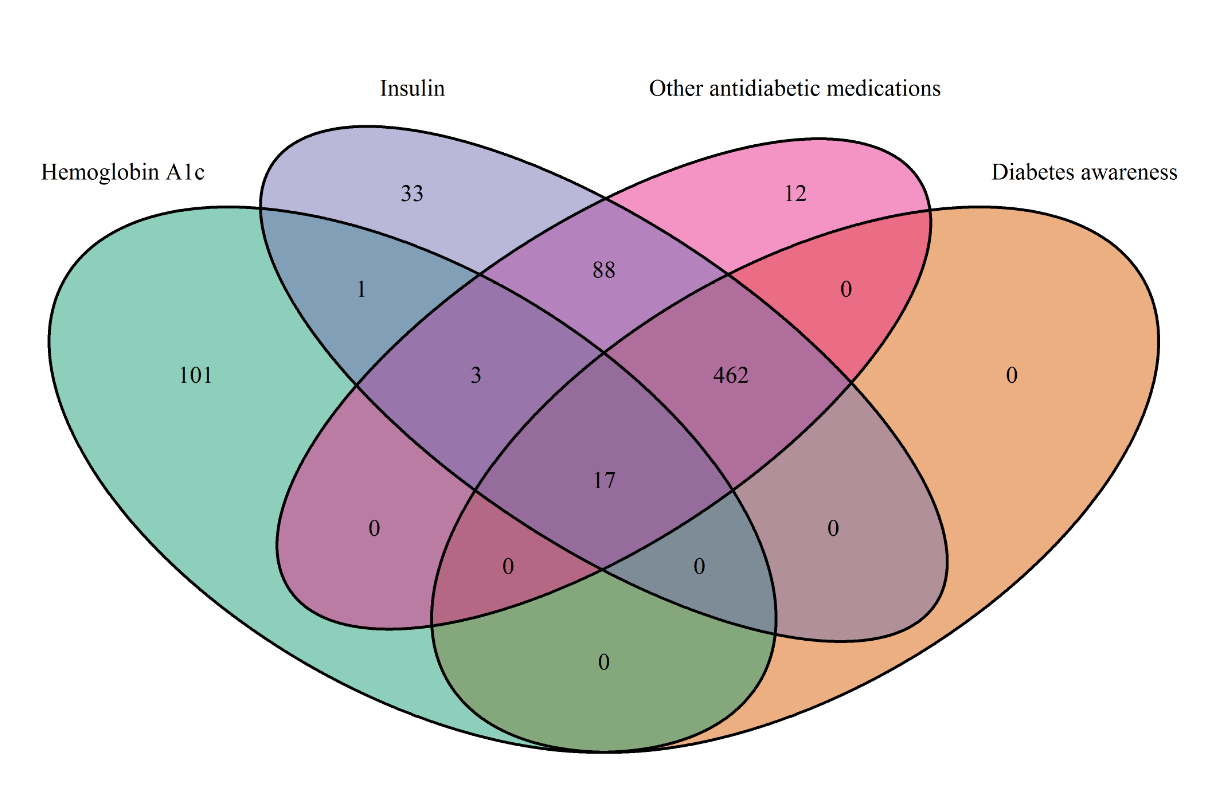

Supplement: Supplementary file 1 — Additional file 1 : Supplementary Table 1. Prevalence of prediabetes, undiagnosed and diagnosed diabetes according to gender and age. Supplementary Table 2. Diagnosed diabetes disaggregated by glycemic control according to gender and age. Supplementary Table 3. Descriptive characteristics of LOFUS participants by glycemic status. Supplementary Table 4. Definitions of diagnostic groups. Supplementary Figure 1. Venn diagram for missing/ unclassified data for one or more diabetes defining factors. [file 12889_2020_9791_MOESM1_ESM.docx]
